# Mapping Network Activity in Sleep

**DOI:** 10.3389/fnins.2021.646468

**Published:** 2021-03-22

**Authors:** Priyattam J. Shiromani, Carlos Blanco-Centurion, Aurelio Vidal-Ortiz

**Affiliations:** ^1^Ralph H. Johnson VA Medical Center, Charleston, SC, United States; ^2^Department of Psychiatry and Biobehavioral Science, Medical University of South Carolina, Charleston, SC, United States

**Keywords:** calcium imaging, sleep, brain networks, hypothalamus, microendoscopy

## Abstract

It was in the influenza pandemic of 1918 that von Economo identified specific brain regions regulating sleep and wake. Since then researchers have used a variety of tools to determine how the brain shifts between states of consciousness. In every enterprise new tools have validated existing data, corrected errors and made new discoveries to advance science. The brain is a challenge but new tools can disentangle the brain network. We summarize the newest tool, a miniature microscope, that provides unprecedented view of activity of glia and neurons in freely behaving mice. With this tool we have observed that the activity of a majority of GABA and MCH neurons in the lateral hypothalamus is heavily biased toward sleep. We suggest that miniscope data identifies activity at the cellular level in normal versus diseased brains, and also in response to specific hypnotics. Shifts in activity in small networks across the brain will help identify point of criticality that switches the brain from wake to sleep.

Waking, non-rapid eye movement sleep (NREM sleep) and REM sleep are hypothesized to be triggered by activity of specific neurons in the brain. A number of mathematical models have been proposed to identify how the neuronal activation may control the switching between the states. One early model based on the Lotka-Voltera equations proposed that a reciprocal interaction between neurons in the brainstem regulated the alternations between sleep-wake states (McCarley and Hobson, 1975). This model was succeeded by the “flip-flop” switch model when it was discovered that there were sleep and wake neurons in the hypothalamus and that these neurons linked with brainstem neurons to regulate wake-sleep transitions (Saper et al., 2010). Another model, referred to as the “two-process model of sleep regulation” incorporates an interaction between the circadian pacemaker (Process C) and a homeostatic element- Process S in the regulation of sleep-wake (Borbely, 1982; Borbely et al., 2016). A new model (Krueger et al., 2008) incorporates glia, which have been overlooked, and hypothesizes that sleep arises from local network activity between glia and neurons.

A common element of these models is that they postulate that the alternation between sleep and wake is regulated by complex interactions between brain cells (Figure 1). There is an interdependence between cells so that when one type of neuron, glia or neurotransmitter is manipulated there is an effect on the others. For instance, deletion of the noradrenergic locus coeruleus neurons or even of three neuronal populations (noradrenergic, cholinergic and histaminergic) forces a reorganization of the daily patterning of sleep-wake architecture (Blanco-Centurion et al., 2004, 2007; Shiromani et al., 2004). How does the brain maintain stability in patterning the daily organization of sleep-wake states? The concept of self-organized criticality has been proposed to show how complex systems maintain stability (Bak et al., 1987). Self-organized criticality is best illustrated in the “sand pile” experiment where the addition of sand grows the pile to a critical point so that each new grain of sand causes shifts, termed avalanches, in the sand pile that maintain the correct slope (Bak et al., 1987). Indeed, small and large shifts in patterns of neural activity in sleep and wake is evidence of criticality (Beggs and Plenz, 2003; Ribeiro et al., 2010; Priesemann et al., 2013; Fontenele et al., 2019), and are considered necessary to maintain the neural state for optimal functioning (Hengen et al., 2013; Hengen et al., 2016; Lombardi et al., 2020). Researchers have tracked cortical activity as evidence of criticality during sleep-wake behavior. However, considering that sleep-wake are generated by subcortical neuronal populations, it is important to track longitudinally the activity of sub-cortical neurons, especially neurons implicated in generating wake, non-REM and REM sleep. Traditionally, standard electrophysiology has been used to identify activity of neurons during sleep and wake (Sakai, 2019). However, the limitations of *in vivo* microwire electrophysiology make it difficult to track neural activity over long periods. To disentangle brain circuits during complex behaviors, such as sleep, requires new technology (reviewed in Shiromani and Peever, 2017). Do shifts in patterns of neural and glial activity anticipate changes in vigilance states?

**FIGURE 1:**
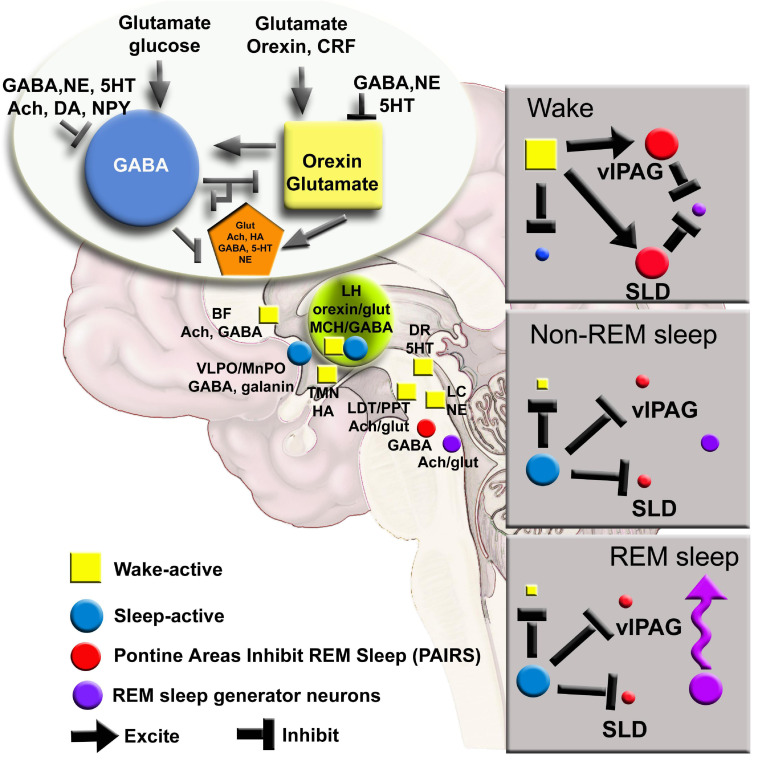
A network map of neurons regulating waking, NREM and REM sleep has been derived from lesion, electrophysiology and c-FOS studies. New tools can further refine the model to include glia and determining how activity in small networks propagates across the brain. Ach, acetylcholine; BF, basal forebrain; CRF, corticotrophin releasing factor; DA, dopamine; DR, dorsal raphe; glut, glutamate; HA, histamine; LC, locus coeruleus; LH, lateral hypothalamus; MnPO, median preoptic area; NE, norepinephrine; NPY, neuropeptide Y; SLD, sublateral dorsal nucleus; TMN, tuberomammillary nucleus; vlPAG, ventral lateral periaqueductal gray; 5HT, serotonin.

This review focuses on live-cell imaging of cells, a method that makes it possible to image activity of individual neurons and glia deep within the brains of freely behaving animals. The method enables real-time monitoring of activity of individual neurons and glia across conditions and over many days to months. From the dataset it is possible to map the activity of the cells, and to test hypotheses regarding normal versus abnormal activity in the network. The network map can be used to identify abnormal activity, and then to restore normal network traffic by repairing defective nodes in the circuit using the gene transfer approach or with optogenetic stimulation.

## The Deep-Brain Imaging Method

**FIGURE 2:**
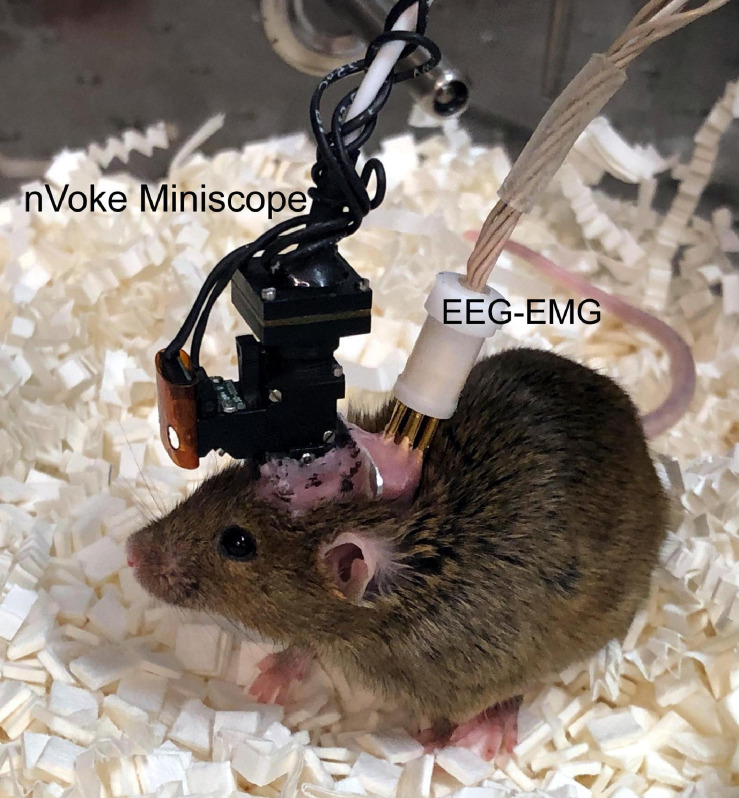
A mouse with an implanted nVoke miniscope (Inscopix.com) and electrodes to record sleep. A single-photon mini-microscope weighing 2 g can be securely attached to the skull along with electrodes to record sleep. The nVoke miniscope enables simultaneous optogenetic stimulation and imaging, allowing investigators to mechanistically determine network activity of specific neurons or glia during sleep-wake states.

Currently, the EEG is used to record activity of the brain, and the EEG machine still represents the cornerstone of all sleep labs. However, the EEG measures activity at the cortical surface but does not reveal anything about activity in individual cells at the sub-cortical levels. A new method has been developed that images activity of individual cells deep within the brains of freely behaving mice and rats (Jennings et al., 2015). The images are captured with a miniature single-photon microscope (Inscopix.com; dbaharoni.com; Labmaker.org; open-ephys.org), also referred to as a miniscope, that sits atop the animal’s head (Figure 2). The miniscope connects to a microendoscope that penetrates the brain. The microendoscope is a glass lens, referred to as a GRIN lens (Gradient-Index; Thor labs), that focuses the images of the underlying cells onto the miniscope. The miniscope captures changes in intracellular levels of calcium (Ca^2+^) (Flusberg et al., 2008; Ghosh et al., 2011). Special genetically tagged sensors, referred to as GCaMP, fluoresce as a function of the rise in intracellular Ca^2+^ levels, and the miniscope is able to capture the change in fluorescence. At rest, the cells containing the GCaMP will exhibit a basal fluorescent signal. However, when a cell (astrocyte or neuron) is excited there is an increase in the levels of calcium inside the cells, causing an increase in the intensity of the fluorescence. The change in intensity of the fluorescent signal can be empirically determined (df/f) and reflects the activity of the cell (Tian et al., 2009). It has been established that the fluorescence is linked to depolarization of the neuron (Chen et al., 2013). In our studies we have confirmed that calcium fluorescence is directly linked to action potentials (please see Blanco-Centurion et al., 2019).

**FIGURE 3:**
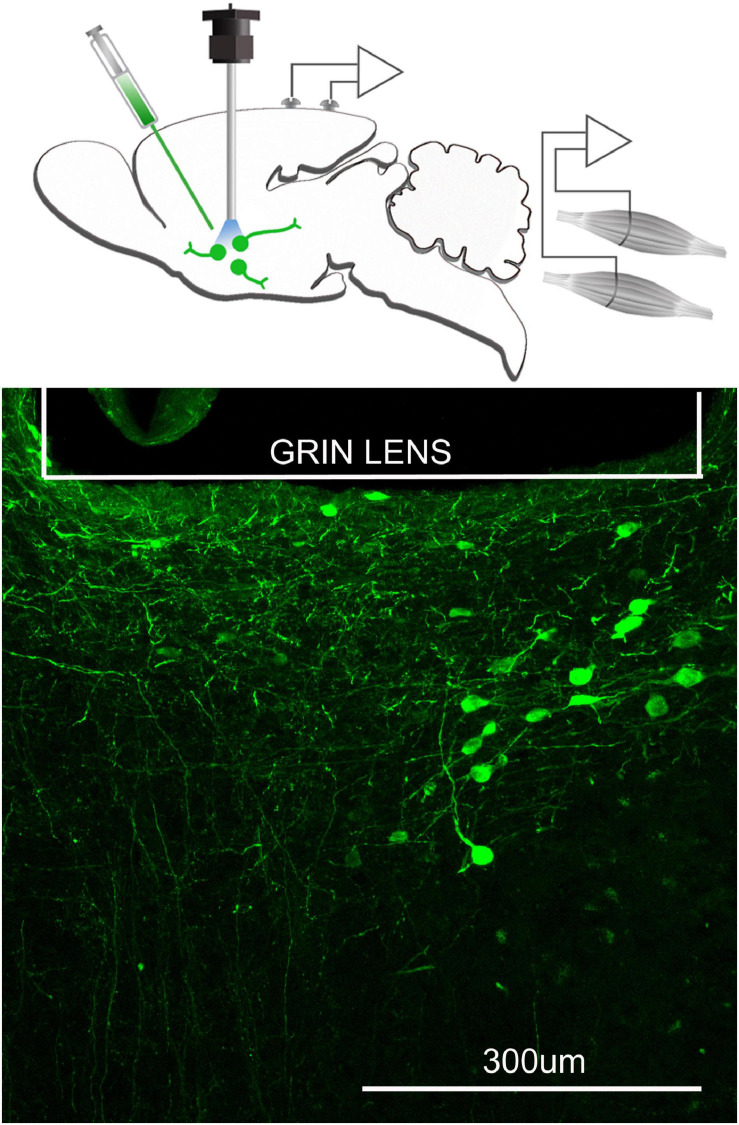
Neurons containing the calcium indicator, GCaMP6, relative to the focal plane of the GRIN lens. The top figure schematically depicts that a microinjection of AAVDJ-EF1a-DIO-GCaMP6m is made to express the calcium indicator, GCaMP6 in neurons that are cre positive. At the same time a GRIN lens (600 μm diameter) is inserted at the injection site and electrodes to record the EEG and EMG are implanted. Three weeks after injection, a miniscope is attached to image the fluorescence associated with the intracellular changes in calcium. Bottom figure is the postmortem histology depicting many GCaMP6 containing neurons below the GRIN lens.

To image a specific phenotype of neurons or glia an adenovirus containing GCaMP (Stanford Neuroscience Gene Vector and Virus Core, University of North Carolina or addgene.com) is injected into target sites in the brain where cells that facilitate Cre-mediated recombination are located (Figure 3). Mice that express Cre in specific phenotypes of neurons or glia are available (Jax.org). The mice are anesthetized (isofluorane; 2–3% continuous gas) and placed in a stereotaxic instrument. The AAV-viral particles are slowly injected into the target site with a microliter syringe (usually 0.2–1 μl volume). A GRIN lens is inserted at the microinjection site and the baseplate cemented onto the skull. At this time EEG and EMG electrodes are attached to the skull and the entire assembly secured with dental cement (please see Blanco-Centurion et al., 2019 for details). It is important to consider where to insert the GRIN lens as it does damage the brain. The mice are returned to their home cages and allowed to recover for 21 days from the surgery. This time period also enables the GCaMP6 to be expressed in the Cre-positive cells at the injection site, and the cells begin to fluoresce as a function of the change in intracellular levels of calcium (Chen et al., 2013).

A typical experiment begins with a 3-day adaptation period that allows the animals to acclimate to the sleep recording cables and the miniscope. During the adaptation period the focusing on the miniscope is adjusted to obtain the sharpest images of the fluorescence in the somata. The focal plane can be adjusted up to 200 μm by manually turning the miniscope. The position of the miniscope that gives the sharpest images is noted and the neurons are imaged at that position. It is possible to adjust the focal plane so that other neurons become visible. This makes it possible to image many more neurons along the dorsal-ventral focal plane.

An experimental session consists of recording sleep and fluorescence images through a number of wake, NREM and REM cycles. The images can be recorded while the animal is engaged in various tasks (feeding, grooming, locomotor activity) over a period of days. Or, the neurons can be imaged during bouts of cataplexy in narcoleptic mice models. Thus, the same neurons can be imaged longitudinally allowing for pre-post experiments. Upon completion of the experimental paradigm, post-mortem histology is done to confirm the presence of the GCaMP6-containing neurons within the focal plane of the GRIN lens (Figure 3).

The data consists of video images captured by the miniscope, and the data are stored on high-capacity storage disks (typically one-terabyte solid-state drives). A high-end computer workstation with a powerful video graphics processor is necessary to analyze the video images. The data processing is done offline using specialized software (Mosaic from Inscopix.com or ImageJ at NIH.gov) that are compatible with MatLab, and allow the data to be exported and integrated with the sleep data (Neuroexplorer.com).

**FIGURE 4:**
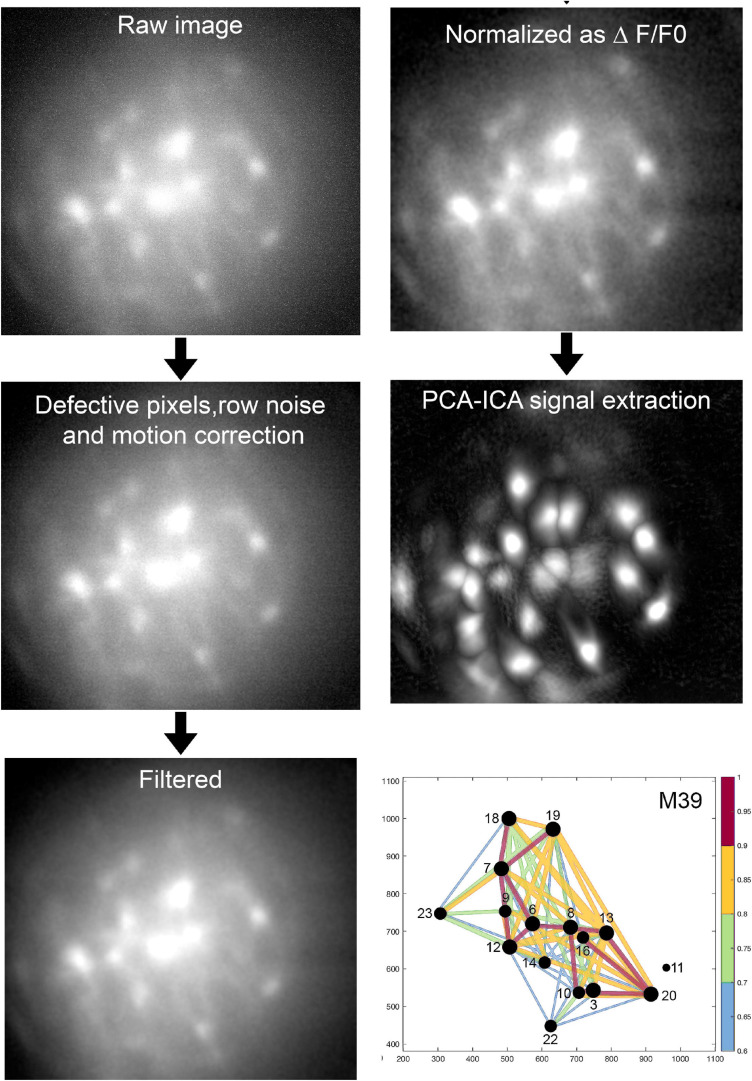
Analysis of imaging data. The above sequential process is followed (Mosaic software) in an unbiased manner and blinded to treatment. Proven algorithms convert the raw data to a network map where the intensity and timing of the fluorescence signal between pairs of neurons is represented by the diameter of the circle and thickness of the line connecting pairs of neurons. For full description of data analysis and statistics please refer to our published paper (Blanco-Centurion et al., 2019).

Figure 4 depicts the sequential stages of data analysis. The raw image file is down sampled (anti-aliasing; 2X), fixed for defective pixels, row noise and isolated dropped frames, if any. The clean images are then corrected for motion artifacts resulting from the animal’s respiration (along the *x, y* axes). If a miniscope is not attached properly then there will be severe motion artifacts and it is best to discard the data. The motion corrected images are further cleaned with a band-pass filter and then a reference fluorescence (F0) frame is determined. All of the frames in the dataset are compared against the reference frame by principal component analysis (PCA) followed by an independent component analysis (ICA). The PCA-ICA analysis yields a defined region of interest (ROI) representing fluorescent cells. If a ROI clearly indicates that it represents two adjacent cells, topographical segregation of calcium signals is achieved by manually drawing the respective ROI. We emphasize that it is necessary to obtain clear fluorescent signals at the time of data collection and this can be done by focusing the miniscope. The investigator will have to conduct preliminary studies to identify the concentration and volume of AAV-GCaMP that yields the clearest signal.

**FIGURE 5:**
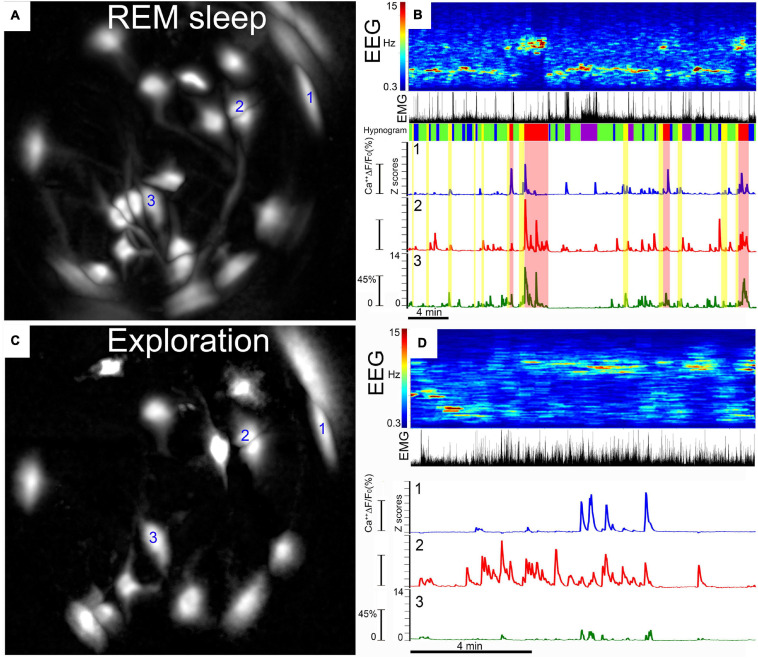
Calcium fluorescence in individual MCH neurons. **(A,C)** Depict the same field of view of the GRIN lens with fluorescence (Δ*F*/*F*0) in somata and processes extracted automatically by PCA-ICA analysis. We have labeled the three neurons (labeled 1, 2, and 3) whose Ca^2+^ fluorescence is plotted in **(B,D)**. **(A)** Depicts GCaMP6s fluorescence (Δ*F*/*F*0) in MCH neurons during REM sleep. Ca^2+^ imaging was performed simultaneously with recording of cortical EEG and EMG activity in the nuchal muscles. Behavioral video recordings were obtained and examined to identify behaviors such as walking, eating, grooming, or eating. Activity in the EEG (depicted as power spectra, 0.3–15 Hz) and the EMG is used to identify wake, NREM, and REM sleep states (labeled as hypnogram). The traces depict the change in fluorescence (Δ*F*/*F*) during wake–sleep bouts of the three neurons identified in **(A)**. In each neuron, the Δ*F*/*F*0 (expressed as a *z*-score) varies with the wake–sleep state of the animal, with peak fluorescence associated with REM sleep. The hypnogram categorizes the sleep–wake states in the following colors: purple, active wake; blue, quiet wake; green, NREM; yellow, pre-REM sleep; red, REM sleep. **(C)** is the same field of view as in **(A)**, but this image shows the PCA-ICA extracted neurons (Δ*F*/*F*0) while the mouse was engaged in exploring novel objects placed in its home cage. This image shows that some neurons that were evident in REM sleep **(A)** were also activated during exploratory behavior. However, some neurons in **(A)** were not evident during exploratory behavior, indicating selective activation of these neurons during REM sleep **(A)**. Thirty percent of the neurons were activated during REM sleep but not during exploratory behavior, indicating that a subset of MCH neurons is selectively active in REM sleep. **(D)** GCaMP6s fluorescence in MCH neurons while exploring novel objects. The traces are from the same neurons represented in REM sleep **(A)**. (For further details, please see Blanco-Centurion et al., 2019).

The change in fluorescence (ΔF) is computed as follows: ΔF = current fluorescence [F] at pixel [*x,y*] minus F0 at pixel [*x,y*] divided by F0 at pixel [*x,y*]. The dataset is normalized (Z scores) and the change in fluorescence in individual ROI is plotted over time (usually in seconds) and correlated with sleep or other behaviors. Figure 5 summarizes the final product of the data analysis. The ROI representing the neurons are clearly visible within the field of view of the GRIN lens (Figures 5A,C). The change in fluorescence in each ROI can be tracked and plotted along with the sleep-wake states (Figures 5B,D).

The same set of cells can be tracked during different conditions or treatments, which allows investigators to determine the activity of the cell over many days to months. Indeed, this a major advantage of live-cell imaging. Electrophysiology can record activity of single neurons but it is extremely labor-intensive, slow and it cannot selectively identify the exact phenotype of the recorded neuron. On the other hand, with live-cell imaging a researcher can now “watch” the activity of individual and populations of neurons of known phenotypic origin and rapidly determine its role in behavior. This method was used to settle the question of the activity pattern of neurons containing melanin concentrating hormone (MCH) (Blanco-Centurion et al., 2019). An electrophysiological study in head-restrained rats had sampled activity of MCH neurons and concluded that the neurons were active only during REM sleep (Hassani et al., 2009). This result remained unchallenged until we used the deep-brain imaging method and found that 70% of the MCH neurons were also active during periods of exploratory behavior during waking (Blanco-Centurion et al., 2019). Another study confirmed the results (Izawa et al., 2019). Activity of glutamatergic (Vglut2-IRES-Cre mice) (Xu et al., 2015), GABA (gad2-IRES-Cre and vGAT-cre mice) (Weber et al., 2015; Cox et al., 2016; Weber et al., 2018; Oesch et al., 2020), galanin (Chen et al., 2018), and neurotensin (Zhong et al., 2019) neurons has been imaged during sleep. Glia have also been imaged during sleep (Bojarskaite et al., 2020; Ingiosi et al., 2020) which will lead to a shift away from the current “neuron-centric” models of sleep regulation.

## Calcium Imaging Identifies Network Activation

The group at UC Berkeley was the first to use the deep-brain imaging method to monitor activity of neurons during sleep (see for example Cox et al., 2016). We were next, and imaged the MCH neurons (Blanco-Centurion et al., 2019). We recently imaged the GABA neurons in the lateral hypothalamus-zona incerta finding that a subset of the GABA neurons anticipated onset of sleep (Blanco-Centurion et al., 2020). We see the utility of brain imaging as a tool to identify network activation of specific circuits (Figure 4). By monitoring network activity in specific regions it is possible to determine the pattern of activity as the sleep-inducing signal propagates across the brain. Such a brain activity map is essential to understanding how the brain shifts between states of consciousness, and what causes sleep disorders. It is now known that a distributed network of neurons generates waking, non-REM sleep and REM sleep (Sakurai et al., 2005; Jones, 2020). The chemical signature and connectivity of these neurons is also known. The next step is to determine the time-course of a signal between these neuronal populations. This will yield a temporal and spatial map of signaling across the brain as the waking brain falls asleep. Which population is affected first? Which population is the last to receive the sleep signal? What is the temporal response of specific sleep versus wake neurons to the stimulation and to the emergence of sleep? What is the temporal response between glia and neurons? Such information is necessary to identify circuits and nodes that are key to sleep. These can then be targeted with hypnotics to facilitate sleep.

**FIGURE 6:**
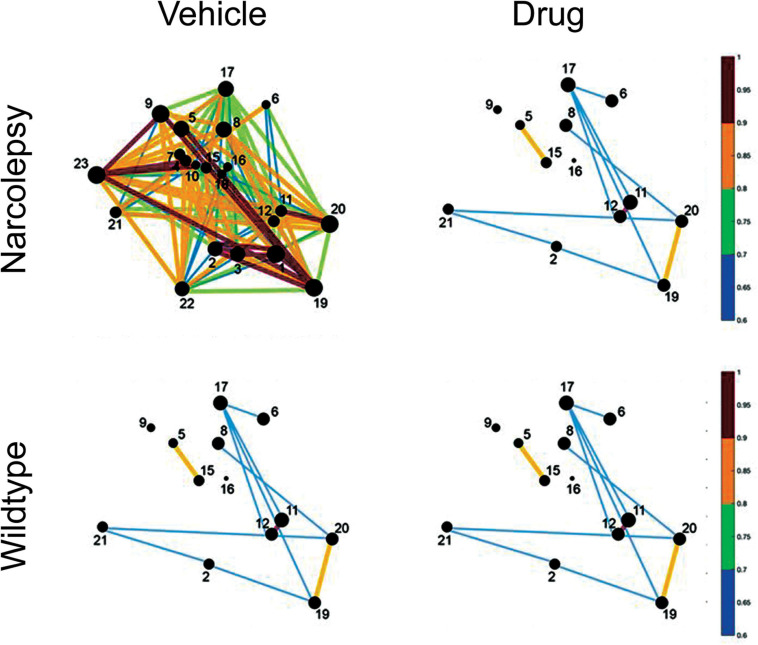
Hypothetical effect of a drug on network activity of sleep-inducing neurons in a mouse model of narcolepsy. The circles represent neurons that increase Ca^2+^ fluorescence during sleep. The size of the circles and the lines between the neurons identifies the strength of the fluorescence between pairs of neurons. In narcolepsy (vehicle) we speculate that perhaps the sleep network is hyperactive which leads to sleep fragmentation and narcoleptic behavior. The drug reduces network activity so that it is similar to that seen in normal wildtype animals.

During the Ebola virus crisis, cell-phone data and activity on social networking sites was used to derive temporal and spatial maps of spread of disease. Indeed, in the current COVID-19 pandemic, cell-phone signals are used to track individuals and the spread of disease. We believe that for the first time it is possible to derive point-to-point activity maps of the brain as it falls asleep. Activity in small networks can differentiate patients in minimally conscious state compared to vegetative state/unresponsive wakefulness syndrome (Demertzi et al., 2015; Di Perri et al., 2016). In other words, functional activity in small networks can identify normal versus diseased brains. Most importantly, pharmacotherapeutics should be able to repair the network and normalize function (see Figure 6). For decades, pharmaceuticals have corrected cardiac, epileptiform and other electrical activities in small networks. However, in the area of sleep disorders medicine there is no data on the effect of pharmacotherapeutics on activity of small sleep networks, even though there are several drugs that are FDA approved for treatment of narcolepsy (Burgess and Scammell, 2012). There is a growing impetus to demonstrate effects of these drugs at the cellular level, first in animal models and then in humans. We argue that any drug demonstrating that it normalizes cellular network activity and function will gain advantage.

The deep-brain imaging approach empowers researchers with the ability to identify activity in neural circuits during identified behaviors. For example, this approach is being used to deconstruct the activity of specific neurons during cataplexy. Activity of GABA (identified with the vesicular GABA transporter in vGAT-cre mice) neurons in the amygdala was imaged in narcoleptic orexin-knockout mice, and it was found that some GABA neurons became active just prior to the onset of cataplexy (Sun et al., 2019). This indicates hyperactivity in a subset of amygdala neurons just prior to cataplexy. The activity of these neurons can be blocked to show that it also blocks cataplexy. Activity of the MCH neurons in the hypothalamus was found to be unchanged indicating that these neurons are not triggering the cataplexy (Sun and Liu, 2020). Indeed, mice with double ablation of the orexin and MCH neurons show more severe cataplexy compared to single deletion of the orexin neurons (Hung et al., 2020).

It is important to recognize that microendoscopy, like electrophysiology, is a tool that provides descriptive data, and other methods, such as optogenetics or chemogenetics, have to be used to mechanistically drive the circuit to cause the behavioral change. The limitation of the calcium imaging method is that it does not reveal rate or pattern of activity of the imaged neurons. For instance, it cannot reveal whether the imaged neurons fired as single spikes or in clusters. It also lacks millisecond precision that is necessary to identify time-course and sequence of events linked to specific behaviors. For example, the calcium signal decay times are 1.5, 1.0, and 0.5 s for the slow, medium and fast types of GCaMP6 (Chen et al., 2013). Moreover, the GCaMP6 fluorescence may quench, which makes it necessary to capture the images for short time periods at a time. However, these limitations are likely to be resolved with faster cameras and more stable dyes.

## Future Directions

Miniaturized electronic amplifiers and battery-powered devices have made it possible to identify sleep in birds during long periods of flight (Rattenborg et al., 2016). A miniature microscope can finally answer questions that have proved to be challenging. The newer miniscopes allow investigators to optogenetically stimulate a cell and image response in the neighboring cell(s). Such a miniscope can stimulate neurons and image activity of adjacent glia, or vice-versa, thereby determining how juxtacellular cells influence each other in freely behaving animals. Microendoscopy can determine the activity of the same phenotype of neurons across divergent species. This may help determine whether REM sleep exists in reptiles and amphibians. We recognize that single-cell electrophysiology studies have been done in monotremes (Siegel et al., 1996, 1998; Siegel et al., 1999) and reptiles (Shein-Idelson et al., 2016) suggesting that REM sleep is present in these animals. However, the question can only be fully settled by monitoring the same phenotypes of neurons that are REM-active across species. Moreover, the miniscope can be used to determine that REM sleep is also unihemispheric, like NREM sleep. This can be done quite easily by imaging fluorescence activity of conserved phenotype of neurons, for example, hypocretin or MCH neurons, in one brain hemisphere and determining whether the fluorescence is directly correlated with desynchronized EEG in that hemisphere.

A major strength of microendoscopy is that it can monitor the same neurons longitudinally over a period of hours, days, or months. As such, the method can determine whether the same neurons and the network become activated during each NREM or REM bout. This would help determine the effect of prolonged waking on network activity. For example, does the network strengthen with sleep loss by recruiting more neurons into the network? Is there a circadian variation in network activity?

## Author Contributions

All authors listed have made a substantial, direct and intellectual contribution to the work, and approved it for publication.

## Conflict of Interest

The authors declare that the research was conducted in the absence of any commercial or financial relationships that could be construed as a potential conflict of interest.
